# Automated Quantification of Brittle Stars in Seabed Imagery Using Computer Vision Techniques

**DOI:** 10.3390/s21227598

**Published:** 2021-11-16

**Authors:** Kazimieras Buškus, Evaldas Vaičiukynas, Antanas Verikas, Saulė Medelytė, Andrius Šiaulys, Aleksej Šaškov

**Affiliations:** 1 Faculty of Mathematics and Natural Sciences, Kaunas University of Technology, Studentu 50, LT-51368 Kaunas, Lithuania; kazimieras.buskus@ktu.edu; 2 Faculty of Informatics, Kaunas University of Technology, Studentu 50, LT-51368 Kaunas, Lithuania; 3 Faculty of Electrical and Electronics Engineering, Kaunas University of Technology, Studentu 50, LT-51368 Kaunas, Lithuania; antanas.verikas@ktu.lt; 4 Marine Research Institute, Klaipėda University, Universiteto 17, LT-92294 Klaipėda, Lithuania; saule.medelyte@ku.lt (S.M.); andrius.siaulys@jmtc.ku.lt (A.Š.); aleksej.saskov@apc.ku.lt (A.Š.)

**Keywords:** underwater imagery, Ophiuroidea, deep learning, semantic segmentation

## Abstract

Underwater video surveys play a significant role in marine benthic research. Usually, surveys are filmed in transects, which are stitched into 2D mosaic maps for further analysis. Due to the massive amount of video data and time-consuming analysis, the need for automatic image segmentation and quantitative evaluation arises. This paper investigates such techniques on annotated mosaic maps containing hundreds of instances of brittle stars. By harnessing a deep convolutional neural network with pre-trained weights and post-processing results with a common blob detection technique, we investigate the effectiveness and potential of such segment-and-count approach by assessing the segmentation and counting success. Discs could be recommended instead of full shape masks for brittle stars due to faster annotation among marker variants tested. Underwater image enhancement techniques could not improve segmentation results noticeably, but some might be useful for augmentation purposes.

## 1. Introduction

Underwater studies are critical from various aspects, such as economic (off-shore wind farms and oil extraction platforms construction), ecological (biodiversity monitoring and impact assessment), and scientific (geology, archaeology, biology studies). The demand for maritime space requires an integrated planning and management approach, which should be based on solid scientific knowledge and reliable mapping of the seabed [1,2]. One of the widely used seabed habitat mapping methods in the continental shelf and deep seas is underwater imagery [3,4]. Technological progress from hand-held cameras to remotely operated vehicles (ROV) and autonomous underwater vehicles (AUV) increases video material amounts and quality. This method’s main advantage is its simplicity, enabling the rapid collection of large amounts of data, and, hence, cost-effectiveness. However, only a small part of the information available in underwater imagery archives is being extracted due to labour-intensive and time-consuming analysis procedures, thus the need for automatic image analysis arises.

Automated solutions should encompass two steps: (1) preparing imagery data by converting video transects into 2D mosaic maps; (2) performing semantic segmentation and quantitative evaluation of seabed coverage. The first step comes from image processing field and is known as photo stitching, while the second step is usually concerned with the application of supervised machine learning. Our work is related to the second step and explores automatic identification and quantification of brittle stars, in 2D mosaics stitched from video material. Semantic segmentation seeks to label each pixel by its corresponding category automatically, and success is usually quantified by the mean intersection over union (mIOU) metric.

Datasets of annotated underwater imagery for semantic segmentation research task are relatively scarce. Some of them appear in coral reef research [5,6], along with a web-based repository and a coral analysis tool named CoralNet [7,8]. However, these images are mainly suited for the task of classification, rather than segmentation. Following the success of deep learning techniques, in which backbone (feature extraction) layers are often pre-trained on ImageNet [9] and later fine-tuned to the streamlined task at hand, [10] similarly exploited CoralNet. A substantial collection of 431068 images with 191 different coral species was used to pre-train the encoder part of DeepLabv3 [11] model, following with a streamlined task of semantic segmentation, improving the mIOU metric from 51.57% to 53.63%. Additionally, the proposed multilevel superpixel strategy for augmenting sparse labels bolstered the mIOU to 76.96% when training with 4193 and testing with 729 images containing 34 coral categories.

Recently, analysis of other categories of species or objects in underwater imagery has also been gaining interest, and authors often experiment with deep learning techniques to achieve pixel-level semantic segmentation with acceptable accuracy. Ref. [12] adapted the DeepLabv3+ [11] model and achieved 64.65% mIOU when training with 2237 and testing with 300 images containing 16 categories (nautilus, squid, plant, coral, fish, jellyfish, dolphin, sea lion, Syngnathus, turtle, starfish, shrimp, octopus, seahorse, person, stone). Ref. [13] introduced an underwater imagery dataset and compared many deep learning models for the semantic segmentation task. Their proposed SUIM-Net model with the VGG-16 backbone achieved 86.97% mIOU when training with 1525 and testing with 110 images containing seven categories (human diver, aquatic plant or sea-grass, wreck or ruins, robot, reef and invertebrates, fish and vertebrates, and sea-floor or rock). [14], instead of using many categories, concentrated on segmenting *Posidonia oceanica* meadows and successfully applied the VGG-16/FCN-8 convolutional architecture and achieved pixel-wise detection accuracy of 96.1% when training on 460 and testing on 23 images. Additional tests on unseen data from other locations and cameras confirmed detection robustness with 94% and 87.6% accuracies.

This work explores brittle stars detection in underwater imagery using deep learning-based semantic segmentation. Two experts annotated brittle stars in seabed mosaics in two variants (full shape and discs only). Several underwater image enhancement methods, most of them from the review by [15], were considered and evaluated as a pre-processing step. The main novelty lies in comparing annotation variants and how switching experts between training and testing affect segmentation accuracy. Additional contribution is evaluating if pre-processing can help the selected seabed mosaics and how accurate the segment-and-count approach is for the brittle stars species.

The article is organized as follows: collection of underwater video material and preparation of 2D seabed mosaics are described in Section 2; methods used for image pre-processing, deep learning model architecture, and post-hoc analysis techniques to count detected objects are outlined in Section 3; experimental results are reported in Section 4; conclusions with some discussion are in Section 5.

## 2. Underwater Imagery

**Figure 1:**
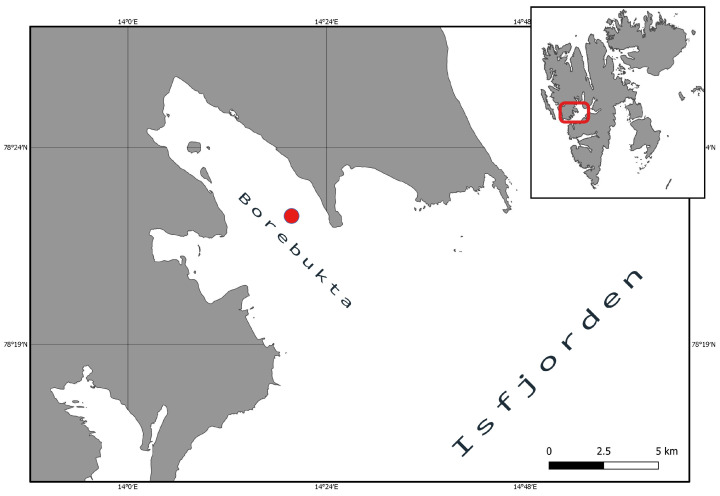
Map of the study area where the seabed imagery was collected. Borebukta bay on Spitsbergen Island, Svalbard (Norway).

Video data for constructing 2D seabed mosaics used in our work were collected in July of 2019 in Borebukta bay on Spitsbergen Island, Svalbard, Norway (see Figure 1). The video was recorded using a remotely operated underwater vehicle (ROV), equipped with a vertically mounted camera (3 CCD, 1920 × 1080 resolution, high-quality Leica Dicomar lenses and 10 × optical zoom) and a lighting system consisting of 16 bright LED in 4 × 4 stations. At a depth of 45 m, approximately 1 m above the seabed, the ROV registered two consecutive 30 s transects.

The raw video transects were later converted into two video mosaics-2D seabed maps, suitable for machine learning training and testing splits. Video mosaicking is a process that involves converting video material into a still image by stitching the overlapping frames. To obtain the dataset used in our experimentations, a video mosaicking method developed by the Center for Coastal and Ocean Mapping (CCOM) [16,17] was used. The process consists of several stages: firstly, a 30 s segment is extracted from a raw video and then compensated for filming platform’s pitch and roll angles, and visually enhanced. The next stage is an automatic frame-to-frame pair-wise registration where the CCOM software calculates neighbouring frames’ overlap. Finally, a 2D mosaic is built by using the overlapping data.

**Figure 2:**
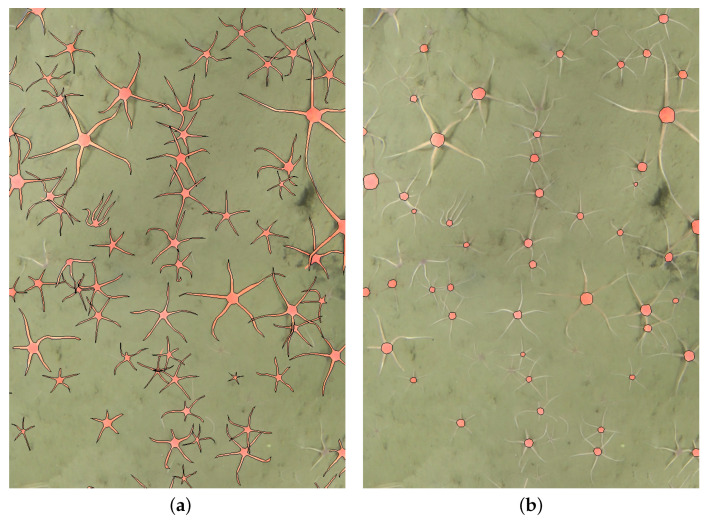
Annotation variants considered when preparing seabed mosaics for the semantic segmentation task. (**a**) Full shape annotation. (**b**) Disc annotation.

The data underlying this article are available in Mendeley Data repository “A fully-annotated imagery dataset of sublittoral benthic species in Svalbard, Arctic” [18,19] where selected 2D seabed mosaics had dimensions of 1487 × 6775 (*Mosaics/B5_0032_30s.jpg*) and 1488 × 7862 (*Mosaics/B5_0102_30s.jpg*) pixels with hundreds of either fully visible or partially hidden brittle stars. Two marine scientists annotated the mosaics in pixel-level detail by drawing closed polygons around visible brittle stars using the online collaborative annotation platform Labelbox [20]. Two variants of annotation were considered: a full star-like shape with tentacles included and a simplified circle-like shape as the main body disc. Example annotations for full and disc shapes are shown in Figure 2.

The prepared dataset consists of two data sources-2D mosaics, referred to as mosaic-1 and mosaic-2, with 361 and 457 markers in full shape or 362 and 500 markers as discs of brittle stars, respectively. One of the marine experts, referred to as expert A, annotated both mosaics in two marker variants (shape and disc). Another expert annotated mosaic-1 only with full shape masks (443 instances). Due to the nature of brittle star positioning on the seabed, it is much easier to correctly annotate brittle star bodies (disc shapes), which explains the disparity between the full vs disc shape instance counts in the mosaics.

## 3. Methods

This section introduces the methods used for mosaic pre-processing through underwater image enhancement, the deep learning model applied, the assessment of segmentation success, and blob count estimation for post-processing.

Fully convolutional network (FCN) [21] is a form of deep neural network that swaps the last fully connected layer, used for the classification task, to convolutional, thus making whole network have only convolutional layers. Extending this modification with some form of upsampling FCN models can be tailored to solve pixel-level classification tasks like semantic segmentation. They have shown to produce favourable results in many computer vision scenarios and even underwater imagery segmentation. It has been shown that through a pyramid pooling module [22] deep neural networks develop the capability of extracting global context information by aggregating region-based contexts. Such architecture is named a pyramid scene parsing network (PSPNet). Once trained, the neural network is tasked with segmenting the seabed mosaics into two classes: brittle stars and background. The resulting segmentation is then used to quantify brittle stars in the region, mainly by denoising the erroneous predictions and using connected component analysis (CCA) to count the brittle star instances.

### 3.1. Image Enhancement

Feasibility of various image enhancement techniques for possible improvements of deep learning model’s segmentation results was tested by experimenting with 2D mosaics enhanced using Python and Matlab implementations of methods reviewed by [15]. The following 13 methods were explored for pre-processing: 4 from underwater image colour restoration and 9 from underwater image enhancement. The main difference between these categories is the use of the optical imaging physical model-the underwater image formation model (IFM) [23], where colour restoration methods are IFM-based and image enhancement methods are IFM-free. IFM seeks to decompose the scene’s colour captured by the camera into the direct transition and background scattering component, which is especially important in artificial lighting conditions. Image enhancement (IMF-free) methods seek to improve the contrast and colour through pixel intensity redistribution avoiding direct modelling of underwater imaging principles but still dealing with water-specific deteriorations such as hazing, colour cast, and low contrast. As summarized by results in [15], the IFM-free methods effectively improve contrast, visibility, and luminance of the underwater imagery but have a downside of unnatural chromaticity and introduced noise.

Underwater image colour restoration methods considered:

1. dark channel prior (DCP) [24];
2. maximum intensity prior (MIP) [25];
3. removal of water scattering (RoWS) [26];
4. Paralenz colour correction [27] (with the gain set to 0.5).

Underwater image enhancement methods considered:

1. contrast limited adaptive histogram equalization (CLAHE) [28];
2. Matlab-based enhancement ensemble (Fusion) [29];
3. gamma intensity correction (GC) [30];
4. integrated colour model (ICM) [31];
5. relative global histogram stretching (RGHS) [32];
6. unsupervised colour correction (UCM) [33];
7. underwater dark channel prior (UDCP) [34];
8. underwater light attenuation prior (ULAP) [35];
9. de-hazing with minimum information loss and histogram distribution prior (TIP2016) [36].

**Figure 3:**
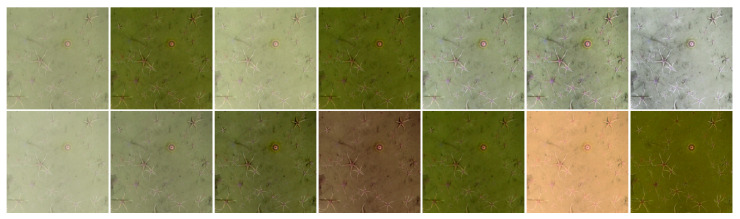
Example result of underwater image enhancement (left to right, starting from the top row): original raw image, DCP, MIP, RoWS, Paralenz, CLAHE, Fusion, GC, ICM, RGHS, UCM, UDCP, ULAP, TIP2016.

Visual examples of image pre-processing results are shown in Figure 3 for qualitative comparison. Instead of quantitative comparison, which was done in [15] by using five objective metrics (entropy, image quality evaluation, etc.), we pre-process full mosaic images and then use them further for training deep learning models and testing accuracy of the resulting segmentation. We assume that such a comparison of segmentation accuracy would help to directly measure the usefulness of restoration and enhancement methods as a pre-processing step for the data selected and task performed.

### 3.2. Deep Learning Model

**Figure 4:**
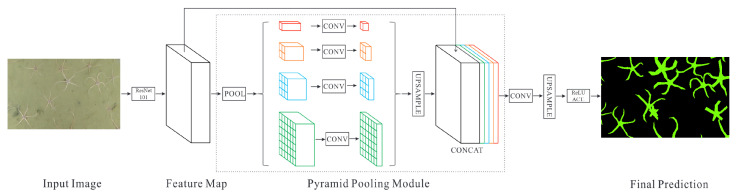
The input to the network is a patch of the mosaic, the output - semantic segmentation mask. Adapted from Ref. [22]. Since the feature map output by the ResNet-101 backbone is 1/8 the input image size, the pyramid pooling module is followed by upsampling through bilinear interpolation to get a segmentation mask of proper dimensions.

For our experiments, we considered a deep convolutional neural network-PSPNet [22] model containing ResNet-101 [37] as a backbone (feature extraction network) with its weights pre-trained on ImageNet. The Keras [38] framework (version 2.3.1), running on Tensorflow [39] backend (version 2.1.0), was used with segmentation-models [40] (version 1.0.1) package. The architecture of the convolutional neural network model used in the context of our work is shown in Figure 4.

**Figure 5:**
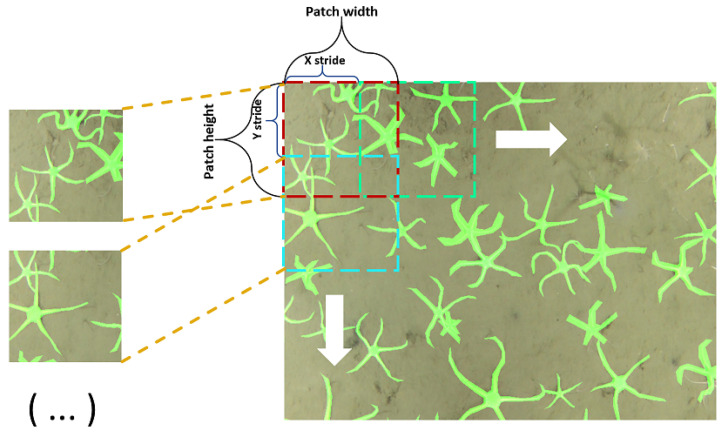
A generalized overview of sliding window approach to slice an input image into patches for training input.

The training parameters for the model are shown in Table 1. The parameter named “patch size” represents the size of the input image in training, “batch size” indicates the number of images used for the weight tuning step, “down-sample” represents the downsampling rate which corresponds to the backbone depth in PSPNet model. The training loss minimized is an additive combination of Jaccard [41] and Focal [42] losses. The model was trained for 500 epochs on full mosaics or 300 epochs on halved ones. The rectangular patch size of 288 × 288 pixels implies that block processing will be required to slice mosaics into patches. The patches were extracted using 144 × 144 strides to slide the patch over the input image. This procedure, also known as sliding window approach, is shown in Figure 5.

By training models with different annotation strategies (disk shape or full shape), we gain insights into segmentation and the subsequent quantification effectiveness.

### 3.3. Segmentation Performance

To evaluate the deep learning model, we have used Intersection-Over-Union (IOU), a common evaluation metric in semantic image segmentation, measuring segmentation success by comparing the ground truth with the prediction mask.

The IOU metric is defined as:

$$\begin{array}{cc} \mathrm{IOU} & =\frac{\mathrm{true}\;\mathrm{positive}}{\mathrm{true}\;\mathrm{positive}\;+\;\mathrm{false}\;\mathrm{negative}\;+\;\mathrm{false}\;\mathrm{positive}\;} \end{array}$$

The IOU metric is obtained from the confusion matrix, calculated using the output threshold of 0.5 for the model’s predictions. Please, note that confusion matrix is not related to object detection task, but to pixel-level assignment of the correct class. Therefore, true positive should be understood here simply as an overlap between prediction and ground-truth, whereas denominator in IOU formula is a union between prediction and ground-truth.

### 3.4. Connected-Component Analysis

To get the most of the segmented mosaic masks, objects, which in our case are brittle stars, ought to be quantified. The standard algorithmic way of achieving this is by performing the connected-component analysis (CCA) [43]. CCA is an algorithmic application of graph theory: given a subset of connected components, each one is uniquely labelled on a given heuristic.

In our work, the workflow to achieve the quantification of objects using CCA is as follows:

1. Reduce the noise in the predicted segmentation mask by morphological opening (erosion followed by dilation).
2. Isolate and remove blobs having an area smaller than the set threshold.
3. Calculate the Euclidean distance transform (EDT) [44] for the smoothed image.
4. Apply the 8-connectivity CCA and perform the watershed transform [45] on the resulting markers.

In our case, the blob count, corresponding to the number of brittle stars, is assumed to be the number of unique labels after applying the watershed transformation step. Expertly achieved parameter values for this workflow are shown in Table 2 (two kernel size and two minimal area values for the disc and full shape segmentations, respectively).

## 4. Experiments

We used the two expertly annotated 2D mosaics block-processed into patches of 288 × 288 pixels with a stride of 144 pixels both for training and testing the neural network model. This processing resulted in 528 image patches for mosaic-1 and 514 patches for mosaic-2. The experiments were conducted to evaluate the effectiveness of deep learning application on individual mosaics and different segmentation markers (full shape vs central disc), assess image enhancements and, finally, evaluate segmentation differences when switching between annotators for training mosaic-1.

### 4.1. Experimental Setup

The hardware configuration was as follows: Intel(R) Core(TM) i7-8700 CPU @3.2 GHz, 32 GB of operating memory, NVIDIA GeForce RTX 2070 with 8 GB of graphic memory. The software configuration was as follows: Windows 10 Enterprise (build 1809) 64-bit operating system, CUDA 10.1, CuDNN 6.4.7 and Python 3.6. The applied model takes approximately 5 s per epoch to train. In all experimental settings, the loss converged after approximately 250 training epochs, but model trained on the preset maximum number of epochs was used for inference.

### 4.2. Experimental Results

**Figure 6:**
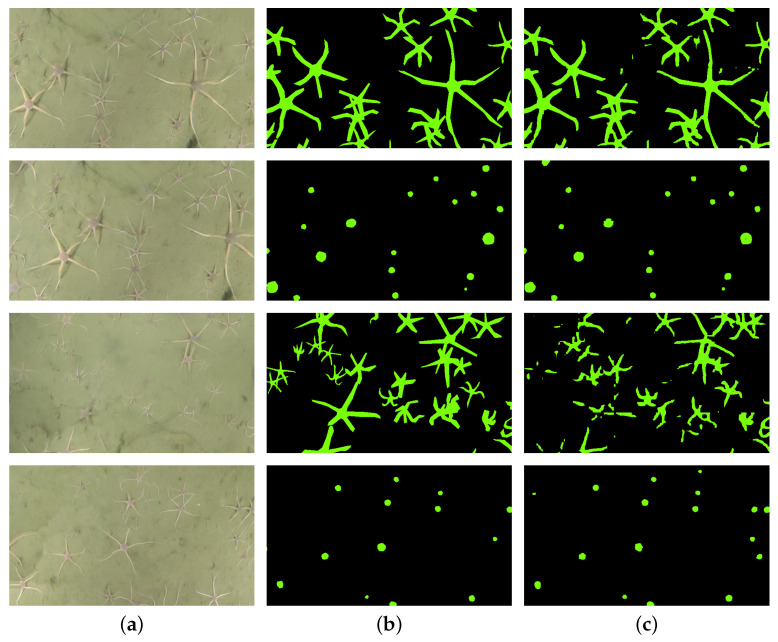
Segmentation results when testing on mosaic-1: comparison between acceptable (*first two rows*) and not so successful (*last two rows*) mask predictions. (**a**) Raw image. (**b**) Ground truth. (**c**) Prediction from PSPNet.

Table 3 reports segmentation performance for different combinations of training and testing mosaics. In case the same mosaic is used both for training and testing, learning is performed on one-half of the mosaic by splitting it horizontally at the middle and training on the top while testing on the bottom halves. When mosaics for training/testing differ, the entire images are used. As seen from the results, the highest IOU score for the experiment with full shape annotations is 58%. The lowest performance score results from training and testing on mosaic-1. Surprisingly, when training on mosaic-1, better results are achieved on the mosaic-2, whereas the same can not be said when training on mosaic-2. For segmentation of brittle star discs, the best-achieved IOU is about 75% when training on the same mosaic. The effect of full shape training does not repeat here: better scores are achieved when training and testing on the same mosaic. Figure 6 shows some examples of better and worse segmentation areas in mosaic-1 for full and disc shape markers.

To better understand the pre-processing effects on the underwater mosaics, enhancements described in Section 3.1 are used to transform the images. The segmentation performance when using this setup is shown in Table 4.

From the reported results in Table 4 it can be seen that none of the enhancements noticeably contribute to better segmentation results, especially when training on mosaic-1 and testing on mosaic-2.

Since two experts annotated mosaic-1, the resulting cross-validation between the annotators might garner useful information. The results are shown in Table 5.

The results show that the segmentation performance decreases when training and testing mosaics with ground-truth masks from different annotators.

Connected component analysis results (shape counts) are shown in Table 6. Despite the achieved mediocre IOU values, the match of estimated counts for brittle stars in the mosaics ranges from 78% to 93% if compared to the annotator’s ground-truth.

## 5. Discussion

Depending on how model performance indicators are evaluated, in a strict or forgiving way, results could differ significantly. The reason for this is not only model performance but also variations in raw material quality. In this study, organisms had moved during recording, which resulted in various artefacts in the mosaics. More artefacts were introduced due to the mosaic stitching process. In some cases, brittle stars were clipped and, in others, multiplied. It significantly affected full shape model performance: in many cases, separated legs in the imagery were confusing even to an expert to annotate, and even more so for the model (see Figure 6). Although central disk annotations and model results also suffered from raw data artefacts, they did so to a lesser degree. Therefore, some of the model mistakes should be interpreted differently.

**Figure 7:**
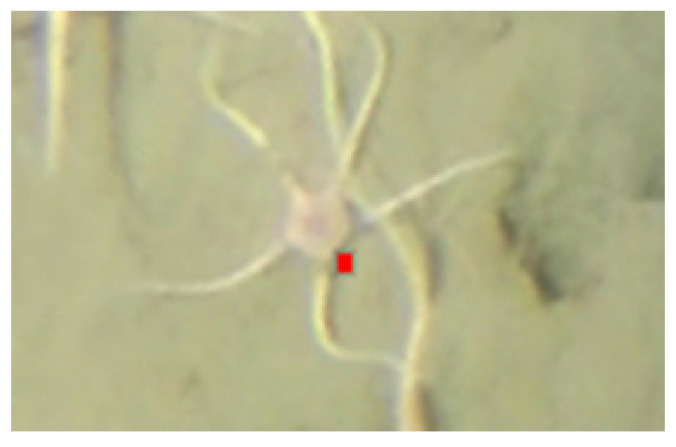
Example of false positive prediction which is still useful for counting.

When the model falsely detected the central disk in some false positive cases, the detected object was still associated with the actual organism (see Figure 7). Therefore even if a central disk was not accurately detected, the count estimate of organisms was correct. Depending on what to consider as the final result, such a detection result could be considered correct or incorrect.

**Figure 8:**
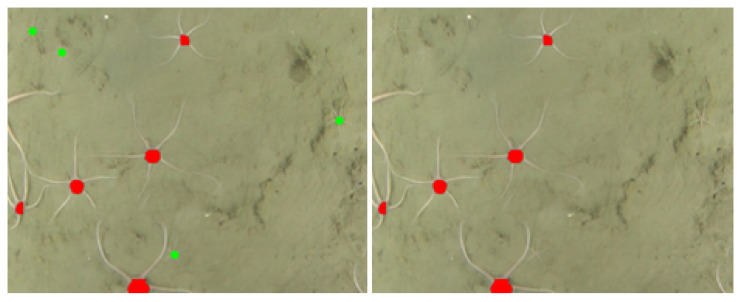
Example of false negative prediction from the model: organisms with hardly visible disk, marked in green color (*left hand side*), were missing in the prediction result (*right hand side*).

Some organism instances in the mosaic were very tiny, although still detectable by a human expert. However, in some such cases, the central disk is not visible in the imagery (see green coloured disks on the left hand side of Figure 8). One cannot expect that model trained to mainly detect clearly visible central disks could easily detect a rare case of brittle stars with no disk visible, therefore, some of the false negatives can be explained not by model errors but by flaws in the scenery. We made two model performance evaluations by a human expert: a strict one not considering the problems described and a forgiving one.

In Table 7, two kinds of evaluations for false positive and false negative are provided: strict evaluation, where predictions were left as is and forgiving, where some cases were excluded from false predictions. For false positives, exclusion occurred when the disk was not correctly detected but the organism was present or expert did not annotate that organism. For false negatives, exclusion occurred when no distinguishable central disk could be observed in imagery but expert still annotated that organism.

For mosaic-1, the forgiving evaluation of disc shapes reduces false positives by 27 (from 45 to 18) and, consequently, increases ratio of correctly counted blobs from 0.944 to 1. Actually, when applying forgiving evaluation the ratio of correctly counted instances exceeded 1 since a few brittle stars were not annotated by the expert. For mosaic-2, forgiving evaluation of disc shapes reduces false positives by 39 (from 82 to 43) and, consequently, increases ratio of correctly counted blobs from 0.786 to 0.864. False negatives were reduced by 2 for mosaic-1 and by 4 for mosaic-2, indicating that organism with its disk hidden beneath the sand was a rare case.

Performance noticeably decreased when training on annotations of one expert and testing on annotations of another expert. Such a drop in performance could be partially explained by different annotation styles when experts were marking tentacle parts-tentacles annotated by the expert A were consistently thicker. This result highlights the importance of discussing the annotation style before starting these labour-intensive efforts when annotations do not overlap. In case of overlapping annotations (when several annotators label the same object), variance in annotations could be exploited to increase amounts of training data as a specific variant of augmentation. Also, merging of annotations, or even devising a survey to vote for the best ground-truth [19], could be considered.

From pre-processing methods explored only RoWS and Paralenz methods were able to marginally improve the IOU values. Surprisingly, the other methods tested seem to negatively affect segmentation performance. In case of training on mosaic-2 and testing on mosaic-1, more methods (6 out of 13) provide a positive effect on the segmentation, although these improvements are rather marginal (difference is less than 0.01 in IOU metric). This lack of improvement from pre-processing could be due to mosaics being very similar in colour and lighting. It is expected that the usefulness of pre-processing could be more pronounced if testing images are more different from training ones with more significant colour mismatches and the problem of domain adaptation.

In the future, we plan to expand this work by increasing the number of examined species from the used fully annotated Arctic imagery dataset with the inclusion of more fauna (e.g., tube-dwelling *Polychaeta*) as well as flora (e.g., brown alga- kelp *Laminaria*) classes. Comparison of PSPNet to other existing deep learning architectures, such as DeepLab [11] or Mask R-CNN [46] or LinkNet [47], with respect to segmentation accuracy and computational efficiency, is of utmost importance. Additional improvements could be related to the model ensemble and efforts to combine several architectures’ outputs into a fused segmentation result. The overall vision of current research would be a collaborative platform for semi-automatic analysis of large and diverse underwater imagery from the Baltic Sea and the Arctic Ocean.

## 6. Conclusions

Extracting valuable information from underwater imagery in the form of segmentation masks and using these masks to count the instances of objects of interest is an important research avenue for benthic studies. For seabed inspection purposes computer vision enables new opportunities to explore and understand the seabed in different regions. An invasive species of brittle star, previously restricted to Pacific ocean, has surprisingly established itself at some places in the Atlantic [48]. Techniques explored here could be useful in measuring abundance of megafauna for commercial or invasive species and quantitatively monitoring organisms such as crabs, scallops, crown-of-thorns sea stars, flatfishes, sea urchins, etc.

The PSPNet based model trained using expertly annotated 2D seabed mosaics (from Svalbard region in Norway) was more successful for segmenting discs than full shapes. Count estimates of extracted blobs corresponded to the ground truth rather well with 78.6–94.4% of brittle stars detected and counted. After forgiving evaluation these estimates increased even more. Therefore, we suggest using disc masks for marking brittle stars, since discs are way more straightforward to annotate. Overall, a relatively low segmentation performance both for full shapes (54.9% and 56.2%) and discs (72.8% and 73.1%) could be not only due to incomplete overlap between prediction and ground-truth, but also due to annotators missing some stars, which could have inflated false positives.

With convolutional neural networks leading in several research areas, deep learning algorithms attracted significant interest in multiple fields due to state-of-the-art achievements. However, these algorithms were unable significantly impact the domain of underwater imaging as of yet, primarily due to the lack of available training data. Instead, various image pre-processing approaches to remove depth-related distortions in underwater imagery are researched in the respective domain. We have found the RoWS method as promising, and although such pre-processing did not provide large improvements for selected imagery, it could be potentially helpful for data augmentation purposes, especially with more varied imagery.

## Figures and Tables

**Table 1:** Summary of deep learning model parameters.

Parameter	Value	Parameter	Value
learning rate	0.00012	dropout rate	0.3
down-sample	8	patch size	288
activation	sigmoid	batch size	8

**Table 2:** CCA workflow parameters.

Parameter	Value
Operator	dilation
Variant	opening
Kernel form	ellipse
Kernel size	(2, 2); (4, 4)
Minimal Area	40 px; 120 px
EDT minimal local distance	55
CCA connectivity	8

**Table 3:** Segmentation performance by the IOU metric for different training/testing configurations.

Train Mosaic	Test Mosaic	Full Shape IOU	Disc Shape IOU
mosaic-1	mosaic-1	0.533	0.744
	mosaic-2	0.562	0.731
mosaic-2	mosaic-2	0.582	0.751
	mosaic-1	0.549	0.728

**Table 4:** The full shape segmentation performance by the IOU metric obtained after pre-processing underwater images by various colour restoration or image enhancement methods. Top 5 results for each column are denoted in bold face.

Method	Train on Mosaic-1,	Train on Mosaic-2,
	Test on Mosaic-2	Test on Mosaic-1
DCP	0.550	**0.552**
MIP	0.553	0.549
RoWS	**0.566**	**0.551**
Paralenz	**0.563**	**0.551**
CLAHE	0.548	**0.553**
Fusion	**0.555**	0.493
GC	0.552	0.547
ICM	**0.561**	0.550
RGHS	0.528	0.548
UCM	**0.557**	0.549
UDCP	0.539	**0.553**
ULAP	0.503	0.548
TIP2016	0.541	0.545
none	0.562	0.549

**Table 5:** The full shape segmentation performance by the IOU metric obtained when switching annotators-training using annotations of one and testing using annotations of another.

Train Mosaic	Test Mosaic	IOU
mosaic-1-A	mosaic-1-B	0.455
mosaic-2-A	mosaic-1-B	0.444
mosaic-1-B	mosaic-1-B	0.549
	mosaic-1-A	0.506
	mosaic-2-A	0.421

**Table 6:** Blob count results after post-processing predicted segmentation.

Annotations	Train Mosaic	Test Mosaic	Blob Counts		
			Blobs Detected	Ground Truth	Ratio
Full shape	mosaic-1	mosaic-2	377	457	0.824
	mosaic-2	mosaic-1	337	361	0.933
Disc shape	mosaic-1	mosaic-2	393	500	0.786
	mosaic-2	mosaic-1	342	362	0.944

**Table 7:** Inspecting disc shape counts for two types of evaluation. Test mosaic `mosaic-1’ means that the model was trained on `mosaic-2’ and vice versa.

Test Mosaic	Strict Evaluation		Forgiving Evaluation	
	False Positives	False Negatives	False Positives	False Negatives
mosaic-1	45	7	18	5
mosaic-2	82	6	43	2

## Data Availability

The data used in this study are available in Mendeley Data repository “A fully-annotated imagery dataset of sublittoral benthic species in Svalbard, Arctic” [18,19].
